# Privacy-Preserved Behavior Analysis and Fall Detection by an Infrared Ceiling Sensor Network

**DOI:** 10.3390/s121216920

**Published:** 2012-12-07

**Authors:** Shuai Tao, Mineichi Kudo, Hidetoshi Nonaka

**Affiliations:** Division of Computer Science, Hokkaido University, Kita 8 Nishi 5, Kita-ku, Sapporo 060-0808, Japan; E-Mails: mine@main.ist.hokudai.ac.jp (M.K.); nonaka@main.ist.hokudai.ac.jp (H.N.)

**Keywords:** behavior analysis, fall detection, privacy-preserved, ceiling sensor network, infrared sensors

## Abstract

An infrared ceiling sensor network system is reported in this study to realize behavior analysis and fall detection of a single person in the home environment. The sensors output multiple binary sequences from which we know the existence/non-existence of persons under the sensors. The short duration averages of the binary responses are shown to be able to be regarded as pixel values of a top-view camera, but more advantageous in the sense of preserving privacy. Using the “pixel values” as features, support vector machine classifiers succeeded in recognizing eight activities (walking, reading, *etc*.) performed by five subjects at an average recognition rate of 80.65%. In addition, we proposed a martingale framework for detecting falls in this system. The experimental results showed that we attained the best performance of 95.14% (*F*_1_ value), the FAR of 7.5% and the FRR of 2.0%. This accuracy is not sufficient in general but surprisingly high with such low-level information. In summary, it is shown that this system has the potential to be used in the home environment to provide personalized services and to detect abnormalities of elders who live alone.

## 1. Introduction

In recent years, human behavior analysis such as person tracking and activity/action recognition has progressed significantly [1–7]. They are becoming indispensable for providing many kinds of personalized services in response to the implicit/explicit demands of users. Due to the rapid development of sensor devices and the downsizing of computers and electronic devices, the research of human behavior analysis is not limited to that by the use of cameras anymore, but also can be realized by many kinds of sensor devices [4–7].

To provide personalized services in daily life, we need to recognize what the activity of individual user is, and to localize where it happens. In other words, activity recognition and localization are both necessary. However, elderly people, even young people, would not be comfortable to be observed for a long time, or to be required any cooperation for giving some information to the systems. In this situation, therefore, there are some important issues we have to concern, e.g., the elimination of disturbance to our daily life or cooperation requirement to the users.

One of the greatest dangers for aged people living alone is falling. More than 33% of people aged 65 years or older have one fall per year [8]. Almost 62% of injury-related hospitalizations for seniors result from falling [9]. Also, the situation will further exacerbate if the person cannot call for help. Therefore, reliable fall detection is of great importance for elders who live alone.

Nowadays, the major fall detection solutions use some wearable sensors like accelerometers and gyroscopes, or help buttons. However, elders may be unwilling to wear such devices. Furthermore, the help button would be useless when the elders are immobilized or unconscious after a fall. Another way of fall detection is to use video cameras. In that case, however, the privacy of the elders is not preserved anymore. They would be uncomfortable to be observed for a long time in the home environment.

To overcome these limitations, in this study, we consider such a system that has little physical or psychological disturbance to our daily life. The sensing devices are supposed to be unnoticeable, and the process of behavior analysis and fall detection is expected to improve the extent of privacy protection of users with respect to cameras. The change of light conditions during the day and at night should not affect the performance. The differences between sensing devices and cameras are summarized in Table 1.

## 2. Related Works

There are many studies about human behavior analysis realized by image processing [10–12]. Moeslund reviews recent trends in video-based human capture and analysis, as well as discussing open problems for future research to achieve automatic visual analysis of human movement [10]. Image representations and the subsequent classification process are discussed separately to focus on the novelties of recent research in [11]. Chaaraoui provides a review on Human Behaviour Analysis (HBA) for Ambient-Assisted Living (AAL) and aging in place purposes focusing especially on vision techniques [12]. Such systems using cameras can always obtain high-level precision of recognition under a suitable light condition, but at home or at office, misrecognizing does not cause a serious problem. Rather, psychological/physical disturbance can be problematic.

Existing solutions for fall detection can be divided into two groups. The first group uses sensors to measure the acceleration and body orientation to detect falls. Some of them only analyze acceleration [13–19]. Lindemann [13] installed a tri-axial accelerometer into a hearing aid housing, and used thresholds of acceleration and velocity to detect falls. Mathie [14] used a single tri-axial accelerometer to detect falls. Prado [15,16] put a four-axis accelerometer at the height of the sacrum to detect falls. The acceleration of falls and activities of daily living (ADLs) were studied in [17]. Especially, it was shown that acceleration from the waist and head were more useful for fall detection than that from wrist. Bourke [18] put two tri-axial accelerometers at the trunk and thigh. Four thresholds were derived and exceeding any of the four thresholds implied an occurrence of fall. In one of our previous works, the speed information was used for fall detection [19]. However, the robustness of the speed thresholds is not sufficient.

Some of them analyze both acceleration and body orientation for fall detection [20–22]. Bourke [20] detected falls using a bi-axial gyroscope sensor based on thresholds. Noury [21] used a sensor with two orthogonally oriented accelerometers to detect falls by monitoring the inclination and its speed. A fall detector consisting of three sensors was developed in [22] to monitor body orientation, vertical acceleration shock and body movements. The common drawback in all these studies is to require the users to wear some sensors. As already stated, many people, especially elders, may feel uncomfortable to wear such devices. There are also some commercial health monitoring products that use a help button to report emergency. However, elders may not be able to do anything after a serious fall. Therefore, automatic fall detection using non-wearable devices is still challenging.

The second group uses video cameras to detect falls [23–25]. An unsupervised method was proposed in [23] for detecting abnormal activity using the fusion of some simple features. In [24], learned models of spatial context are used to detect unusual inactivity. Williams used a distributed network of smart cameras to detect and localize falls [25]. By using video cameras, however, the privacy is easily violated. At least, some people would feel uncomfortable to be observed for a long period.

Another important fact is that, images including the users may not be obtained occasionally due to the existence of obstacles such as tables, sofas and chairs. To overcome this occlusion problem, some researchers [24,26] mounted the camera on the ceiling. Lee [26] detected falls by analyzing the shape and the 2D velocity of the person. However, the privacy-preservation problem has not been resolved yet. In our study, we consider a fall detection system that imposes as little physical or psychological disturbance as possible to our daily life. It is desired that the sensing is unnoticeable from users and the process of fall detection preserves their privacy.

We use a ceiling sensor network of infrared sensors to analyze human behaviors and to detect falls. Twenty infrared sensors were installed on the ceiling in a corner of a lab room as an experimental environment. The novelty of this study is that we regard this 4 *×*5 sensor network as a “top view camera” that has a very poor resolution in principle: 20 pixels with binary values/levels. To increase the intensity level, we take a short-duration average of observed binary values at each pixel. To increase the spatial resolution, we take an expectation over positions of active sensors. In this paper, on the basis of those “pixel values”, eight activities are recognized. In addition, such a technique is further applied to fall detection of a single person.

## 3. System

**Figure 1.**
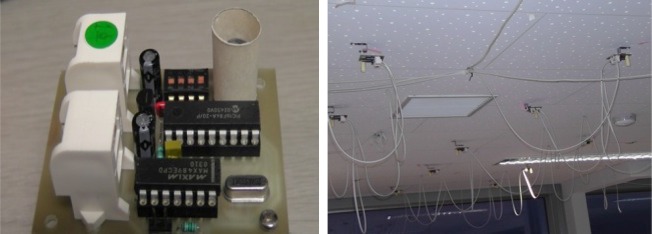
The sensor module and the interconnection of sensor nodes with cables.

In the simulated home environment, we attached “pyroelectric infrared sensors”, sometimes called “infrared motion sensors”, to the ceiling [27]. This sensor detects an object with a different temperature from the surrounding temperature. The photographs of the sensor module and the interconnection of sensor nodes with cables are shown in Figure 1. Such infrared motion sensors are easy to set up at a low cost. The light condition does not affect the performance. Thus, this system can be used in the day and at night.

**Figure 2.**
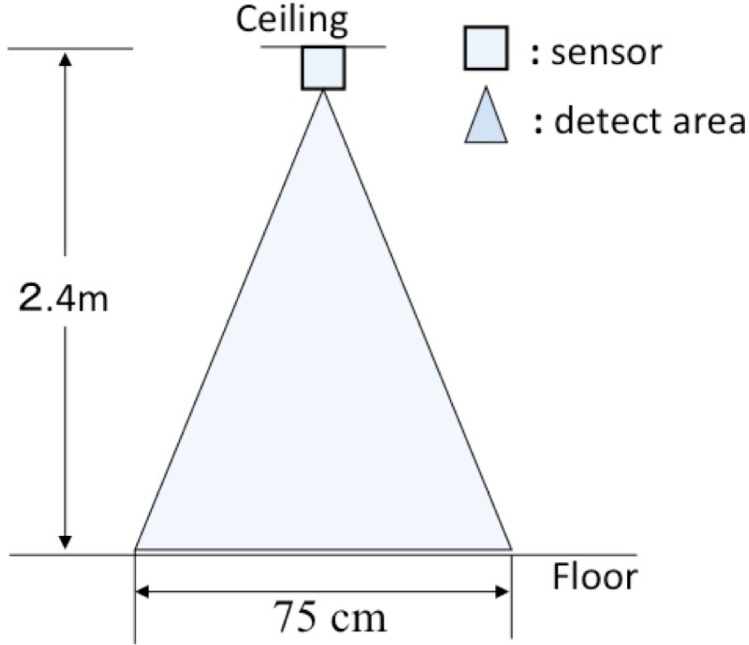
Side view of the detection area adjusted by a paper cylinder.

A hand-made cylindrical lens hood with diameter of 11 mm and length of 30 mm was used to narrow the detection area of each sensor (shown in Figure 1). We set the detection distance of each sensor to 75 cm, from which we can guarantee that a moving person can be detected all the time. The side view of the detection area of a sensor adjusted by the paper cylinder is shown in Figure 2.

**Figure 3.**
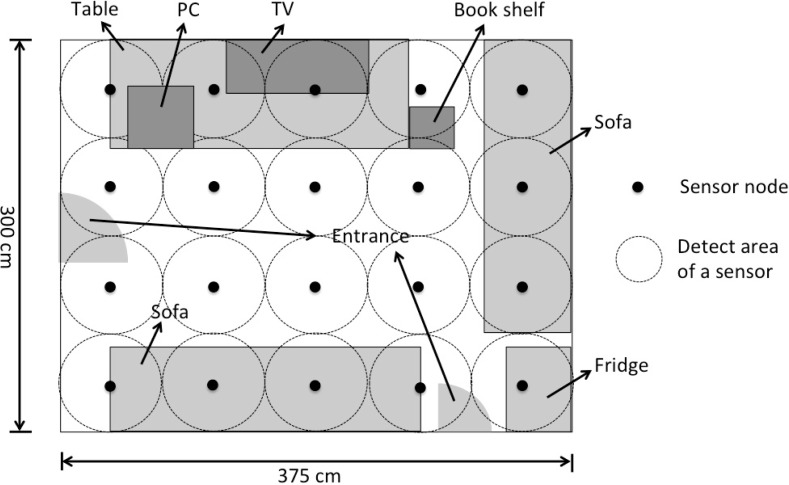
Layout of the home environment and the infrared sensors (top view).

In this study, we rearranged the sensor layout of this system in order to simulate a small room. The twenty sensors were attached to the ceiling (300 cm *×* 375 cm) so as to cover all the area and not to produce any dead space. The average distance between sensors is 75 cm. Figure 3 shows the layout and the arrangement of the sensors. A data collection system was built by C++ program in this study to collect the sensor values. A binary response from each sensor can be read at a sampling rate chosen from 1 Hz to 80 Hz.

There are some characteristics of this sensor equipment room. A moving person often makes multiple sensors active at the same time. To the contrary, the sensor sometimes cannot be active if the person is motionless or moves only slightly, such as when reading a book or watching TV. Therefore, when there is no active sensor, we assume that the person has been staying at the previous position without moving.

## 4. Sensor Network as a Low-Resolution Camera

The infrared ceiling sensor system simply produces 20 (4 *×* 5) binary values at a sampling. We regard our sensor system as a “top view camera” and the sensor responses as a “top view image.” The basic specification of this virtual camera is the resolution of 4 *×* 5 pixels with 2 sensitivity levels. Our basic idea is to increase the sensitivity by accumulating the binary values over a short duration, that is, by lengthening the exposure time of the virtual camera.

Let *s_i,j_*(*t*), (*i* = 1, ⋯, 4, *j* = 1, ⋯, 5) denote the sensor active status (0 or 1) of the sensor locating at (*i, j*) at time *t*. When the sampling rate is *H* (Hz), we define the “pixel value” *p_i,j_*(*t*) at time *t*(> *H/*2):

(1) $$p_{i,j}(t)=\frac{1}{H+1}\sum_{u=t-H/2}^{t+H/2}s_{i,j}(u)$$

It is clear that *p_i,j_*(*t*) ∈ [0, 1]. That is, we take the average of binary responses over one second around time *t*. If a person stays near location (*i, j*) for a long time with a noticeable large motion, the corresponding pixel value *p_i,j_* takes a large value close to one.

A moving person can make multiple sensors active according to his/her moving speed. Therefore, we can estimate the current location of the moving person from the sequence of active sensors.

We suppose that there are *N* (≤ 20) active sensors at time *t*, and they are indicated by their location indices (*i, j*). Let their pixel values be *p_i,j_*(*i* = 1*, ⋯,* 4; *j* = 1*, ⋯,* 5). Then under the assumption that only a single person is in the room, we estimate the location of that person at time *t* by the weighted average as:

(2) $$(x_{t},y_{t})=\frac{\sum_{i=1}^{4}\sum_{j=1}^{5}\left(i,j)p_{i,j}(t\right)}{\sum_{i=1}^{4}\sum_{j=1}^{5}p_{i,j}(t)}$$

**Figure 4.**
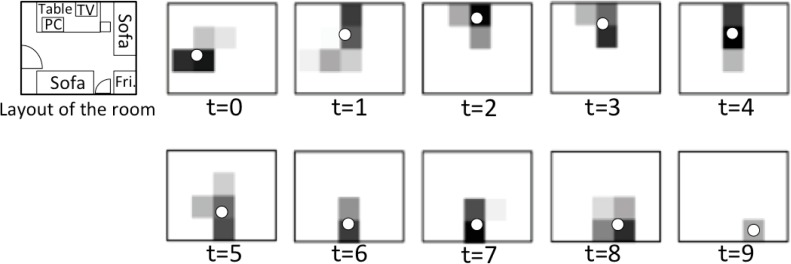
Top view image sequence of a series of activities (the duration of walking, tidying the table, sitting on sofa, switching TV programs, leaving the room). Each image is selected in every two seconds. The gray level corresponds to the pixel value (darker is higher), each white dot shows the estimated position by Equation (2) at time *t*.

**Figure 5.**
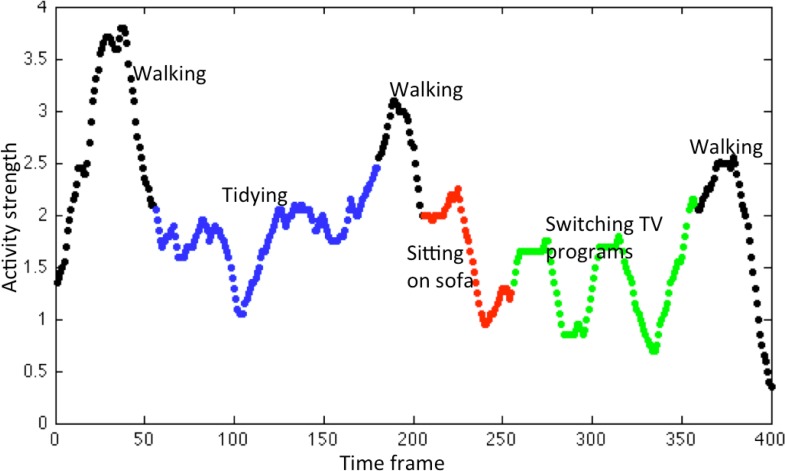
Activity strength (the sum of the 20 pixel values) of a series of activities in the home environment. Different colors show different activities.

We empirically evaluated the accuracy of Equation (2). A subject was instructed to do a series of activities in the home environment (walking, tidying the table, sitting on sofa, switching TV programs, leaving the room, in this order) during about 20 s. We set the sampling rate to *H* = 20 (Hz) to collect the data of *s_i,j_*(*t*). Some of the “top view images” are shown in Figure 4. An example of varying sum of the 20 pixel values, $\sum_{i=1}^{4}\sum_{j=1}^{5}p_{i,j}(t)$, is shown in Figure 5. The value of sum can be thought as the degree of the strength of activities.

In Figure 4, we see that the trajectory of a moving person can be almost captured successfully. By accumulating binary values from a short duration, we enhanced the intensity level of sensors spreading over [0,1] (Equation (1)), and by taking the weighted average of the positions of active sensors, we succeeded to improve the spatial resolution (Equation (2)). Figure 5 shows that we can distinguish to some extent different activities from the average intensity.

## 5. Behavior Analysis

**Figure 6.**
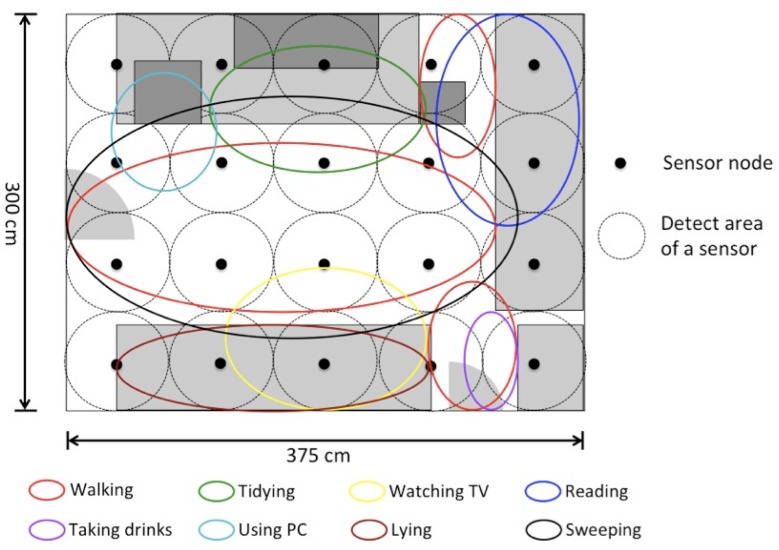
Areas associated with each activity. Different colors show different activities.

In this experiment, we recognized different activities using the “pixel values”. The examined activities of daily living (shortly, ADLs) include “walking around”, “tidying the table”, “watching TV on the sofa”, “reading books on the sofa”, “taking drinks from the fridge”, “using a PC”, “lying on the sofa” and “sweeping the floor”. Each activity can be associated with a specific location (sensing area) as shown in Figure 6, though some locations overlap largely to the others. The subjects are five students belonging to our laboratory (four males and a female). We divided the 5 sets of “pixel values” of five subjects into 4 for training and one for testing. As a result, the average recognition rate was calculated by a 5-fold cross-validation. The classifier was a support vector machine (SVM) with a radial basic kernel with default parameter values. The ground truth was given manually from a video sequence recorded by a video camera that is used for reference only.

**Figure 7.**
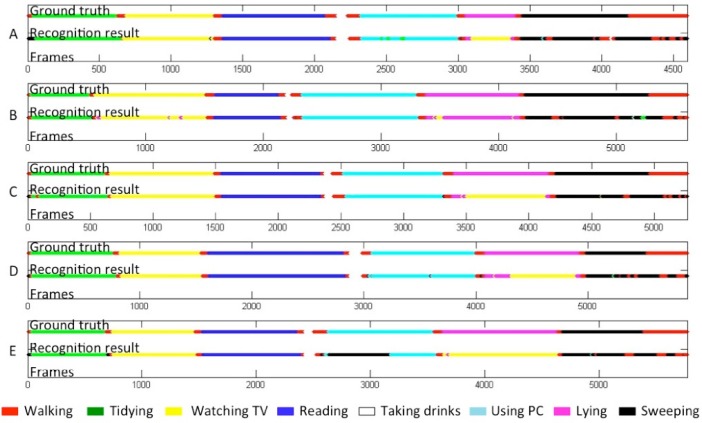
The ground truth and recognition results of five users spending 4–5 minutes in the detection area. Different colors show different activities.

Four different sets of features, *F*_1_–*F*_4_, were examined. The results are shown in Table 2. The largest feature set *F*_4_ including time-difference information was most useful for the recognition and brought a recognition rate of 80.65%. Table 3 shows the confusion matrix of eight activities. The ground truth and the recognition results using *F*_4_ are shown in Figure 7.

In Table 3, the element of row *a* and column *b* indicates the rate that activity *a* was recognized as activity *b*. We see that most of the “lying” are misrecognized to “watching TV” at 68.39%. The reverse-way misrecognition (“watching TV” to “lying”) is seldom seen probably due to the imbalance of data amount. Such a large amount of error is mainly because these two activities share the same location (bottom two ellipsoids in Figure 6). On the contrary, “walking around” and “sweeping the floor” are not so confused (confusion rates of 33.65% and 12.11%, respectively) even though they share a large part of the same location. One possible reason is that there is a difference on speed, so that time difference information included in *F*_4_ contributed to distinguish them.

## 6. Fall Detection

### 6.1. Martingale Framework

Detecting a fall in our system is carried out on the basis of the changes of pixel values. The processing speed to realize online detection is a requirement to be achieved. Therefore, in our study, we use a martingale framework to detect falls from a stream of pixel values [28].

Before we introduce the martingale framework for fall detection, we describe first a fundamental building block called the *strangeness measure*, which assesses how much a data point is different from the others. In the situation of fall detection, the steaming data is unlabeled and thus the strangeness of data points is measured in an unsupervised manner. Given a sequence of vectors of pixel values *P_t_* = {𝕇(1), 𝕇(2), ⋯, 𝕇(*t*)}, 𝕇(*t*) = (*p*_1, 1_(*t*), ⋯ *, p*_4, 5_(*t*)), the strangeness *s_t_* of the current vector 𝕇(*t*) with respect to the previous series of vectors *P_t_* is defined by

(3) $$s_{t}=\left\|𝕇(t)-\text{c}\right\|$$

where **c** is the cluster center, that is, $c=\frac{1}{t}\sum_{u=1}^{t}𝕇(u)$, and || · || is the Euclidean distance.

Using the strangeness measure described above, a martingale, indexed by ∊ ∈ [0, 1] and referred to as a *randomized power martingale*[29], is defined as

(4) $$M_{n}^{\left(∊\right)}=\prod_{t=1}^{n}\left(∊{\hat{q}}_{t}^{∊-1}\right)$$

where the *q̂_t_*’s are computed from the *p̂*-value function

(5) $${\hat{q}}_{t}(\{\text{P}(1),\text{P}(2),\cdots ,\text{P}(t)\},\theta_{t})=\frac{\#\{r:s_{r}>s_{t}\}+\theta_{t}\#\{r:s_{r}=s_{t}\}}{t}$$

where *s_r_* is the *strangeness* measure at time *r* defined in (3), where *r* = 1, 2*, ⋯, t*, and *θ_t_* is uniformly and randomly chosen from [0, 1] at every frame *t*. The initial martingale value is set to $M_{0}^{\left(∊\right)}=1$, and ∊ is set to 0.92 according to the reference [28].

In the martingale framework for fall detection, when a new frame is observed, hypothesis testing takes place to decide whether a fall occurs or not, under the null hypothesis *H*_0_ “no fall” against the alternative *H*_1_ “a fall occurs.” The martingale test continues to operate as long as

(6) $$0<M_{n}^{\left(∊\right)}<\lambda$$

where λ is a positive real number that a user specifies. The null hypothesis *H*_0_ is rejected when $M_{n}^{\left(∊\right)}\geq \lambda$, noticing a “change.” Then a new martingale starts with $M_{0}^{\left(∊\right)}=1$.

Since {*M_n_* : 0 *< n <* ∞} is a nonnegative martingale and *E*(*M_n_*) = *E*(*M*_0_) = 1, according to the Doob’s Maximal Inequality [30], we have

(7) $$P\left(\underset{k\leq n}{\text{max}}M_{k}\geq \lambda \right)\leq \frac{1}{\lambda }$$

for any λ *>* 0 and *n ∈ ℕ*.

It means that it is unlikely for any *M_k_* to have a high value. The null hypothesis is rejected when the martingale value is greater than λ. In Equation (7) is an upper bound for the false alarm rate (FAR) for detecting a fall when there is actually no fall. The value of λ is, therefore, determined by the value of acceptable FAR. For example, we may set λ to 20 if we need FAR lower than 5% as a rule of thumb.

The fall detection algorithm is shown as follows.

**Fall detection algorithm**. Martingale Test (MT)

Initialize: *M*(0) = 1; *t* = 1; *P_t_* = {}.

Set: λ.

1: **loop**
2:   A new frame of 20 pixel values 𝕇(*t*) is observed.
3:   **if** *P_t_* = {} **then**
4:     Set strangeness of 𝕇(*t*): =0
5:   **else**
6:     Compute the strangeness of 𝕇(*t*) and data points in *P_t_*.
7:   **end if**
8:   Compute the *p̂*-values *q̂_t_* using (5).
9:   Compute *M*(*t*) using (4).
10:   **if** *M*(*t*) *> λ* **then**
11:     **FALL DETECTED**
12:     Set *M*(*t*)=1;
13:     Re-initialize *P_t_* to an empty set.
14:   **else**
15:     Add 𝕇(*t*) into *P_t_*.
16:   **end if**
17:   *t* := *t* + 1;
18: **end loop**

### 6.2. Experiment and Results

Since falls are not normal activities seen in our daily life, we asked the subjects to pretend them. In an investigation of the fall of elders, Wei [31] found that 85.0% of the falls are during walking, and 62.5% of the falls happen indoors. Therefore, we set up a virtual “room” in a corner of the laboratory (Figure 3) and asked the subjects to simulate falls in the middle of walking.

In this experiment, a subject was asked to stay in the room for about one minute every round. During this period, the subject behaved naturally and did some of activities randomly such as walking, tidying a table, watching TV, sitting on a sofa, reading books, or taking drinks form a fridge. The subject was also instructed to behave sometimes fall-like activities such as sitting fast and lying on the sofa. After a series of activities, the subject simulated a fall during walking. In total, three of subjects performed 65 normal activities, 20 fall-like activities and 50 true falls.

**Figure 8.**
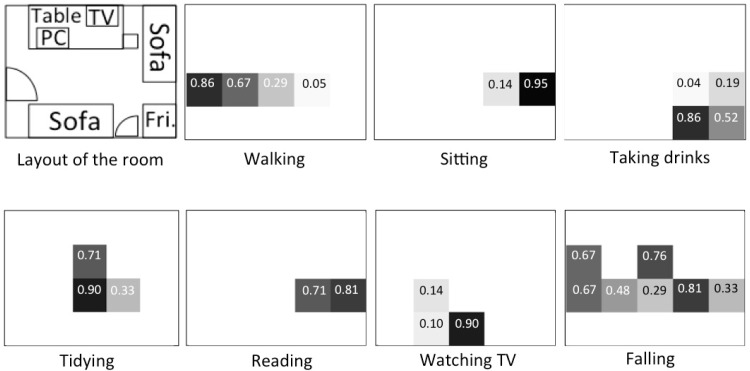
The variation of pixel values when a subject performs some activities. The gray level corresponds to the pixel value (darker is higher), the decimal numbers are the pixel values.

**Figure 9.**
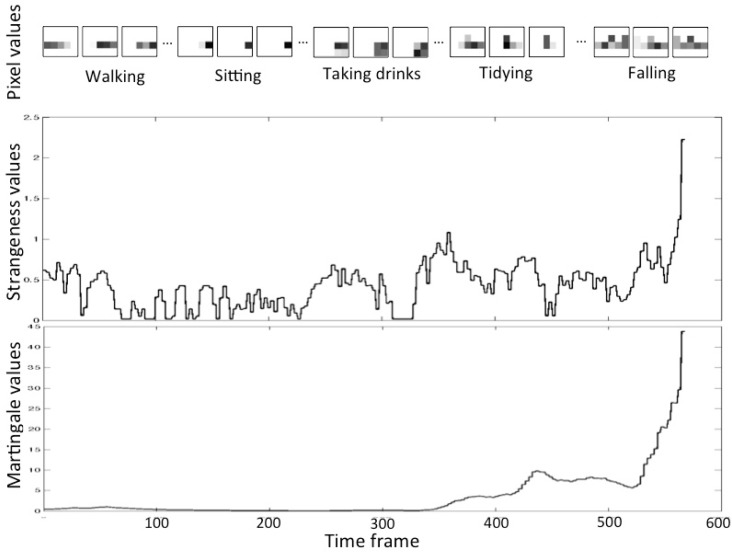
The variation of the pixel values, *strangeness* values and *martingale* values in a series of activities.

Figure 8 shows the variation of pixel values when a subject performed several activities containing a fall. Activities performed sequentially were segmented manually. In the pixel values, we see that the (simulated) fall is different from other activities: fall’s pixel values spread widely. We suppose that the walking speed of an elder is about 1–1.5 m/s. Due to the characteristic of delay of our infrared sensor (a moving person makes multiple sensors active at the same time), when the person is walking before falling, there will be 2–4 active sensors. The number of active sensors depends on the speed and location of the person (below one sensor or between two sensors), which can be seen in Figures 4 and 8. When the person falls after walking, the spread area will be larger due to the stretch of the body, the active sensors will be more (usually 5–8 active ones). Accordingly, the *strangeness* of the pixels of fall is distinct from those of other activities. Figure 9 describes the variation of pixel values, *strangeness* values and *martingale* values in a series of activities in detail. If the pixel values have a large variation in a short time, then the *strangeness* value increases and the martingale value increases as well.

The performance evaluation of fall detection is made based on two pairs of retrieval performance indicators, (precision and recall) and (false alarm rate (FAR) and false reject rate (FRR)). They are defined as

(8) $$\begin{array}{c} \mathrm{Precision}=\frac{\mathrm{Number of Correct Detections of Falls}}{\mathrm{Number of Detections of Falls}},\\ \mathrm{Recall}=\frac{\mathrm{Number of Correct Detections of Falls}}{\mathrm{Number of True Falls}}, \end{array}$$

(9) FAR=1−Precision,FRR=1−Recall.

In addition, we use a single performance indicator *F*_1_ defined as

(10) $$F_{1}=\frac{2\times \mathrm{Recall}\times \mathrm{Precision}}{\mathrm{Recall}+\mathrm{Precision}},$$

representing a harmonic mean between precision and recall. A high value of *F*_1_ ensures reasonably high precision and recall.

**Figure 10.**
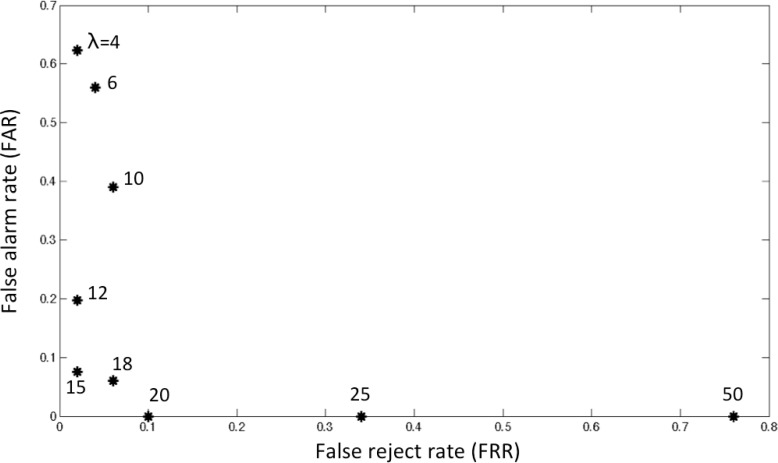
The ROC evaluation for different λ’s.

Table 4 shows the details of the performance in precision, recall and *F*_1_ for several values of λ. The receiver operating characteristic (ROC) evaluation is also shown in Figure 10. In *F*_1_, we attained the best performance of 95.14% at λ = 15, which corresponds to FAR of 7.5% and FRR of 2.0% (Figure 10). It is serious to miss true falls, so we investigated the attainable minimum value of FRR. We can see in Figure 10 that 2.0% of FRR at λ = 15 is the minimum. This corresponds to one case missing among fifty falls. In this case, immediately before overlooking the true “fall”, one “lying down” was misdetected as a “fall” due to its high martingale value and thus a newly started martingale could not detect the succeeding true fall. In contrast, all cases of 7.5% (=4 false alarms/53 detected falls) were fall-like activities. Unfortunately, we could not have a lower value of FRR even if we change the value of λ due to the above-mentioned special case.

**Figure 11.**
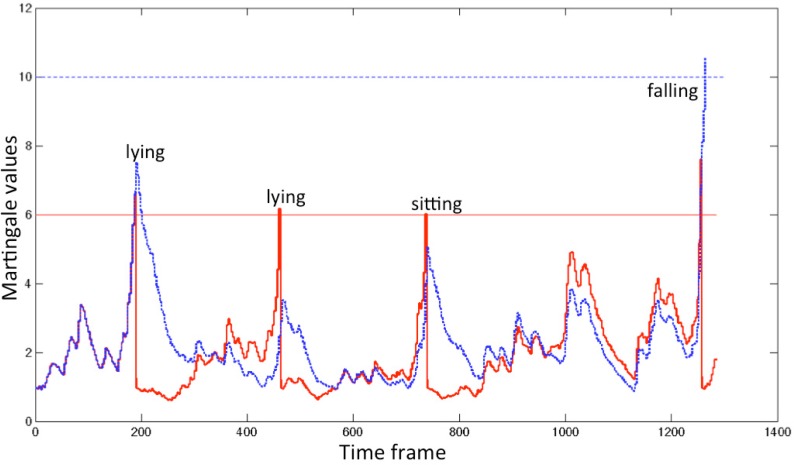
The martingale values when λ is set to 6 (red line) and 10 (blue line).

One example of detection is shown in Figure 11 for λ = 6, 10. By increasing the value of λ from 6 to 10, we can dismiss all false alarms.

## 7. Discussion

In this study, an infrared ceiling sensor network was used to recognize multiple activities and to detect falls in a home environment. Since the sensor system is installed on the ceiling, it is almost unnoticeable by the users. It does not require any cooperation from the users. Different from camera systems, the performance of our sensor system is not affected much by obstacles or light conditions. Most importantly, the privacy of users is always preserved.

However, in the practical usage, there are some limitations in our system. Our classification method relies on the assumption that a distinct activity has its own associated location where the activity is performed. Indeed, many activities are often associated with different locations, e.g., we have a rest sitting on the sofa, take drinks from a fridge and fall asleep in bed. This study basically aims at detecting such location-associated activities. Therefore, different activities carried out in the same location can be detected but it is difficult to distinguish them. However, such confusion usually does not cause a serious problem for ADL recording. Maybe we can combine such activities into one activity.

The system also utilizes the strength of activities, the pixel values, the area and speed information, the number of active sensors and time information from one time step before and after, to improve the performance on classification. These pieces of information make it possible to distinguish two activities even if they share largely their associated locations, e.g., “walking around” and “sweeping the floor.” The same information, especially the spread information of active sensors, brought a high level of detection performance of falls. On the contrary, if the amount is not sufficient, for example, in such cases that a person lies down on a sofa or falls from a fixed position by dizziness or unconsciousness with slight motion, it is difficult to generate sufficient *strangeness* information when he/she falls, our system may not detect the fall. This behavior of the system is sometimes right and sometimes not. In the current system, the sensitivity is controlled by the value of λ.

This system is supposed to be used by the users who live alone, which means that if there are multiple persons in the room, or even there is a pet like a cat or a dog with the user, this system has to be improved to cope with such complicated situations.

The ceiling sensor system is also a little inferior in detection capability of vertical moves due to the ceiling attachment. Therefore, it cannot detect vertical falls in high precision, although such a case is rare compared with forward/backward falls. To compensate the disability, more kinds of devices such as a depth camera could be used with this system.

## 8. Conclusions

In this research, we have developed a ceiling sensor system to recognize multiple activities and to detect falls in the home environment. The infrared sensors output binary responses from which we know only the presence/absence of a user. However, the privacy of users is preserved to some extent and no user cooperation is required in this system. The novelty of this study is that the definition of “pixel values” makes the sensor network work like a top view camera but improving the extent of privacy protection with respect to cameras. The experimental results showed that this system can recognize eight activities and detect abnormalities (falls) both at acceptable rates. The accuracy is not sufficient in general but surprisingly high with such low-level information. This privacy-preserved system has the potential to be used in the home environment to provide personalized services and to detect falls and other abnormalities of elders who live alone.

## Figures and Tables

**Table 1.** Differences between cameras and sensing devices.

**Item**	**Sensing devices**	**Cameras**
Place to use	Anywhere in a room	Indoor and outdoor
Recognize accuracy	Lower	Higher
Number of users	Small	Large
Privacy protection	Strong	Weak
Light condition	No special condition	Stable light
Obstacle condition	Movable obstacles	No obstacles
Establishment cost	Low and flexible	High and fixed

**Table 2.** Examined feature sets and recognition rates.

No. Feature set (no. of features)	Expression	Description	Rec. rate
*F*_1_ Pixel values (20)	*p_i,j_*	Pixel values from 20 sensors	80.41%
*F*_2_ Sum (1)	∑*_i,j_ p_i,j_*	Sum of all pixel values	28.75%
*F*_3_ *F*_1_+*F*_2_ (21)	*p_i,j_*, ∑*_i,j_ p_i,j_*	Pixel values and the sum	73.04%
*F*_4_ Three frame pixel values (60)	(*p_i,j_*)*_t−_*_1_, (*p_i,j_*)*_t_*, (*p_i,j_*)*_t_*_+1_	Pixel values at times *t −* 1*, t, t* + 1	80.65%

**Table 3.** A confusion matrix between eight different activities by feature set *F*_4_.

	Walking	Tidying	Watching TV	Reading	Taking drinks	Using PC	Lying	Sweeping
Walking	53.37	2.57	1.68	3.04	1.80	2.55	1.34	33.65
Tidying	1.09	98.21	0	0	0	0.10	0	0.59
Watching TV	0.85	0	97.69	0	0	0	1.27	0.19
Reading	0.22	0	0	99.78	0	0	0	0
Taking drinks	5.22	0	0	0	94.78	0	0	0
Using PC	0.87	2.01	0	0	0	85.14	0	11.98
Lying	1.11	0	68.39	0	0	0	30.05	0
Sweeping	12.11	0.92	0.24	0	0.31	0.55	0	85.87

**Table 4.** The performance in precision, recall and *F*_1_ for several values of λ.

λ	4	6	10	12	15	18	20
Precision (%)	37.69	43.96	61.04	80.33	92.45	94.00	100.00
Recall (%)	98.00	96.00	94.00	98.00	98.00	94.00	90.00
*F*_1_ (%)	54.44	60.31	74.02	88.29	95.14	94.00	94.74
